# Dynamic alterations in cardiac autonomic regulation during recurrent generalized seizures

**DOI:** 10.1016/j.isci.2026.117048

**Published:** 2026-08-03

**Authors:** Anqi Du, Bo Li, Guangqi Chen, Na Wang, Chuanyu Li, Shaowen Tian, Weichao Liu, Junqing Sun

**Affiliations:** 1 Guangxi Key Laboratory of Brain and Cognitive Neuroscience, School of Basic Medical Sciences, Guilin Medical University, Guilin, Guangxi 541199, China; 2 Guangxi Key Laboratory of Environmental Exposomics and Entire Lifecycle Health, School of Public Health, Guilin Medical University, Guilin, Guangxi 541199, China; 3 Key Laboratory of Medical Electrophysiology of Ministry of Education, Institute of Cardiovascular Research, Department of Cardiology of the Affiliated Hospital, Southwest Medical University, 1 Xianglin Rd, Luzhou, Sichuan Province 646000, China

**Keywords:** epilepsy, cardiac autonomic regulation, heart rate variability, generalized seizures

## Abstract

Epileptic seizures are associated with cardiovascular disturbances, yet how cardiac autonomic regulation changes across repeated seizures remains unclear. Here, using a pentylenetetrazol-induced murine model, we combined ECG telemetry, heart rate variability (HRV) analysis, pharmacological intervention, optical mapping, and brainstem patch-clamp recordings to characterize seizure-associated autonomic responses. Acute seizures produced transient bradyarrhythmias and increased HRV indices that were attenuated by muscarinic blockade. In contrast, repeated seizures reduced HRV and decreased the excitability of cardiac vagal neurons in the nucleus ambiguus, whereas optical mapping detected no significant intrinsic myocardial electrophysiological abnormalities. Together, these findings identify dynamic, stage-dependent alterations in cardiac autonomic regulation during recurrent generalized seizures, with distinct autonomic changes observed following acute and repeated seizure exposure.

## Highlights

• Recurrent seizures produce stage-dependent alterations in cardiac autonomic regulation
• Acute seizure-associated cardiac responses are sensitive to muscarinic blockade
• Repeated seizures reduce cardiac vagal neuron excitability
• No intrinsic myocardial electrophysiological abnormalities were detected

## Graphical abstract

**Figure:**
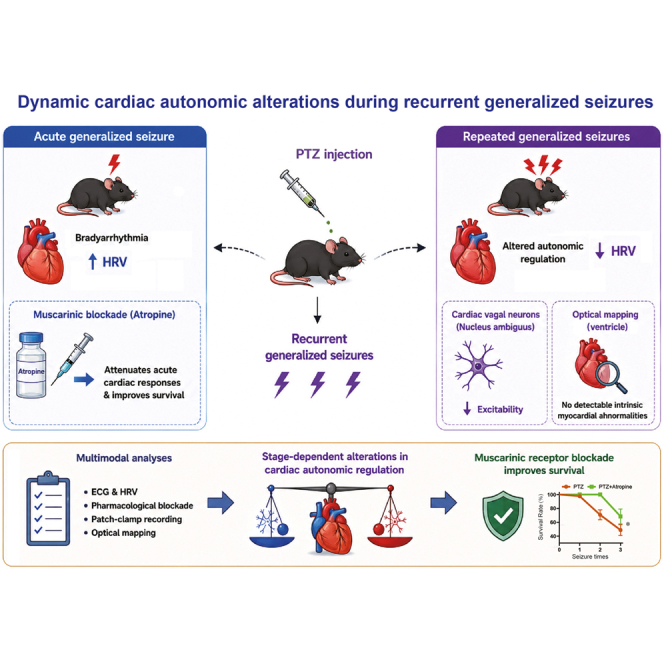

## Teaser

Cardiovascular medicine; Biological sciences; Physiology; Neuroscience

## Introduction

Epileptic seizures are increasingly recognized as systemic events that can disrupt not only brain activity but also peripheral physiological systems, including cardiovascular function. Clinical studies have documented peri-ictal cardiac abnormalities such as bradycardia, asystole, and altered heart rate variability (HRV), especially during generalized tonic-clonic seizures (GTCS).^1^^,^^2^^,^^3^ These disturbances are thought to be associated with seizure-related alterations in autonomic regulation, yet the underlying mechanisms and their evolution over time remain incompletely understood.

Sudden unexpected death in epilepsy (SUDEP) represents a severe clinical outcome in epilepsy and is frequently associated with generalized convulsive seizures.^4^ Postmortem and video-EEG monitoring studies, such as the MORTEMUS study, suggest that fatal events often involve a cascade of cardiorespiratory dysfunction, with respiratory failure typically preceding cardiac arrest.^5^ While these observations highlight a link between seizures and autonomic compromise, SUDEP is a multifactorial process, and the specific contribution of altered cardiac autonomic regulation remains difficult to define in clinical settings.

Clinical investigations of autonomic function in epilepsy have yielded inconsistent findings. Some studies report reduced HRV, suggesting impaired autonomic regulation,^6^ whereas others report differing patterns of autonomic imbalance.^7^^,^^8^ These discrepancies may reflect differences in seizure type, disease duration, and experimental conditions, and underscore the need for controlled experimental models to dissect how seizures influence autonomic regulation over time.

The central autonomic network integrates brain-heart interactions, and alterations within this network have been associated with seizure-associated cardiovascular dysfunction.^2^^,^^9^^,^^10^^,^^11^ However, how seizure-associated changes in central autonomic neurons relate to cardiac outcomes remains incompletely understood. Approaches that combine systemic physiological monitoring with the direct interrogation of identified cardiac vagal neurons (CVNs) are needed to bridge this gap.

Animal models provide an opportunity to examine seizure-associated physiological responses under defined conditions. While genetic models replicate specific epilepsy syndromes,^12^ chemically induced models such as repeated pentylenetetrazol (PTZ) administration enable the study of recurrent generalized seizures and their cumulative effects on systemic physiology.^13^ Although PTZ-induced seizures do not reproduce all aspects of human epilepsy, they reliably generate generalized convulsive events and allow investigation of seizure burden as a driver of physiological adaptation.

In this study, we used a PTZ-induced kindling model in mice to systematically examine how acute and repeated generalized seizures affect cardiac function and autonomic regulation. Employing a multimodal approach that combines ECG telemetry, HRV analysis, systemic muscarinic blockade, optical mapping of cardiac electrophysiology, and whole-cell patch-clamp recordings from identified CVNs in the nucleus ambiguus, we aimed to: (1) determine whether seizure-associated cardiac disturbances are associated with dynamic changes in cardiac autonomic regulation; (2) characterize how these autonomic changes evolve with increasing seizure burden; and (3) evaluate whether seizure-associated cardiac abnormalities are accompanied by intrinsic myocardial electrophysiological alterations. Our findings identify dynamic, stage-dependent alterations in cardiac autonomic regulation across acute and repeated seizures. Acute seizure-associated cardiac responses were sensitive to muscarinic blockade, whereas repeated seizures were associated with reduced HRV and decreased excitability of CVNs. No significant alterations in intrinsic myocardial electrophysiology were detected.

## Results

### ECG changes during seizures

Mice received intraperitoneal PTZ injections (40 mg/kg) three times per week on alternate days. After multiple injections, approximately half of the mice progressed to Grade V seizures, while the remaining animals did not reach this stage. To assess seizure-associated cardiac changes, telemetry devices were surgically implanted on the day after the first Grade V seizure. Following 24 h of postoperative recovery, ECG recordings were performed to assess cardiac responses during subsequent Grade V seizures. A 10-min baseline ECG was recorded prior to PTZ administration, followed by a second 10-min recording initiated 2 min after PTZ injection to capture seizure-associated cardiac alterations. Eight PTZ-treated mice (*n* = 8) that developed Grade V seizures were included for ECG and HRV statistical analysis.

**Figure 1:**
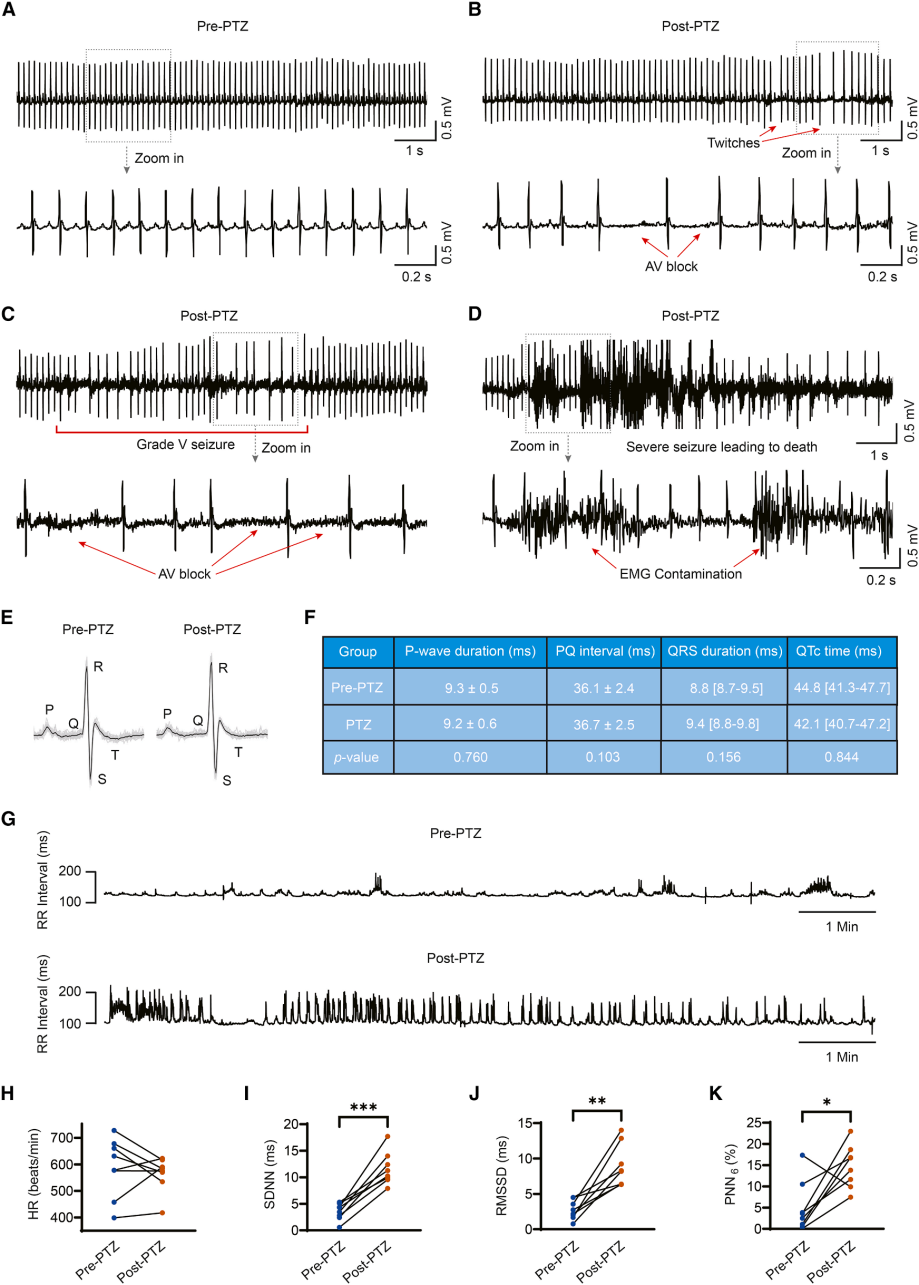
Seizure-associated HRV alterations and cardiac autonomic regulation (A) Pre-PTZ: ECG signal before PTZ administration. No arrhythmias were observed, and the ECG remained stable. (B) PTZ phase: Representative ECG waveforms showing short-term heart rate irregularities and occasional atrioventricular (AV) block during early seizure-related muscle twitches. Zoomed-in view demonstrates the presence of regular P, QRS, and T waves in sinus beats, while non-sinus beats were excluded based on the absence of P waves or irregular R–R intervals. (C) Grade V seizure: Representative ECG showing prolonged rhythm disturbances, including recurrent AV block and transient cardiac arrest during behaviorally defined Grade V motor seizures. (D) Fatal seizure: ECG recorded during a terminal seizure, showing erratic and disorganized cardiac rhythm leading to complete asystole. EMG contamination during convulsions was excluded from HRV analysis. (E) Representative ECG waveforms from the same mouse before and after PTZ injection, showing preserved P, QRS, and T waves despite seizure activity. (F) Quantification of ECG conduction parameters before and after PTZ administration. P-wave duration and QRS duration were analyzed using the Wilcoxon signed-rank test, while PQ interval and QTc time were analyzed using paired t-tests. No significant differences were observed in any parameter (*p* > 0.05 for all; *n* = 8), indicating that no significant changes were detected in the measured ECG conduction parameters following seizures. (G) Representative R–R interval plots illustrating HRV in mice without PTZ (top) and with PTZ-induced seizures (bottom). Seizures led to pronounced beat-to-beat variability. (H–K) Quantification of heart rate and HRV parameters before and after PTZ administration in the same mice. (H) Heart rate (beats per minute) did not differ significantly. (I–K) Time-domain HRV analysis: SDNN (I), RMSSD (J), and PNN_6_ (K) were significantly increased following seizures, indicating increased heart rate variability during seizures. Data are expressed as mean ± SEM or median [IQR], depending on data distribution. Statistical significance was assessed using paired t-tests or Wilcoxon signed-rank tests, as appropriate (*n* = 8). HRV indices were evaluated with Bonferroni correction for multiple comparisons (adjusted α = 0.0167). ∗*p* < 0.05, ∗∗*p* < 0.01, and ∗∗∗*p* < 0.001.

As shown in Figure 1A, ECG signals remained stable before PTZ administration, showing consistent sinus rhythm without arrhythmias. Following PTZ injection (Figure 1B), short-term rhythm irregularities were observed around the time of early seizure-related muscle twitches. These transient disturbances, which were clearly identifiable on the ECG and not attributable to muscle artifacts, were characterized by occasional atrioventricular (AV) block and irregular R–R intervals.

During Grade V seizures (Figure 1C), rhythm disturbances became more pronounced and prolonged, including recurrent AV block and transient episodes of cardiac arrest. These cardiac rhythm abnormalities were observed during behaviorally defined Grade V motor seizures. However, the data do not distinguish among potential contributions from autonomic, respiratory, and peripheral cardiac factors. In fatal seizures (Figure 1D), the ECG showed erratic and disorganized rhythms leading to complete asystole. Electromyographic (EMG) contamination during convulsions was excluded from HRV analysis.

Representative averaged ECG waveforms from the same mouse before and after PTZ administration are shown in Figure 1E. To evaluate whether seizure activity affected cardiac conduction, we quantitatively analyzed ECG parameters (Figure 1F). Statistical analysis revealed no significant differences in P wave duration, PQ interval, QRS duration, or QTc interval before and after PTZ-induced seizures (*p* > 0.05 for all). P wave duration and QRS duration were analyzed using the Wilcoxon signed-rank test, while PQ interval and QTc time were analyzed using paired *t*-tests. These results indicate that no significant changes in the measured ECG conduction parameters were detected following PTZ-induced seizures.

To assess seizure-associated changes in autonomic regulation, 10-min ECG recordings were analyzed for HRV. Representative R–R interval plots before and after PTZ administration are shown in Figure 1G. Baseline heart rate (HR) did not differ significantly between pre-PTZ (589 ± 40 bpm) and post-PTZ (564 ± 23 bpm) groups (*p* = 0.461, paired *t* test; Figure 1H). However, HRV indices increased significantly following seizures (Figures 1I–1K). The standard deviation of normal-to-normal intervals (SDNN) increased from 3.5 ± 0.6 ms to 11.6 ± 1.1 ms (*p* < 0.001, paired *t* test, power = 1), the root-mean-square of successive differences (RMSSDs) increased from 2.7 ± 0.5 ms to 8.9 ± 1.1 ms (*p* < 0.01, paired *t* test, power = 0.99), and the percentage of normal-to-normal intervals differing by more than 6 ms (PNN_6_) increased from 4.9 ± 2.1% to 14.7 ± 1.8% (*p* = 0.014, paired *t* test, power = 0.79). HRV indices remained statistically significant after Bonferroni correction for multiple comparisons (adjusted α = 0.0167). These findings are consistent with increased beat-to-beat variability during seizures and suggest a shift in autonomic regulation. However, given the limitations of HRV in the presence of seizure-related rhythm disturbances, these changes should be interpreted in conjunction with pharmacological and electrophysiological evidence.

### Effects of systemic muscarinic blockade on seizure-associated HRV changes

To examine whether muscarinic signaling contributes to seizure-associated cardiac responses, atropine was administered systemically before PTZ challenge. A 10-min baseline ECG was recorded, followed by intraperitoneal atropine administration (0.5 mg/kg). After a 10-min waiting period, PTZ (40 mg/kg) was administered, and a second 10-min ECG recording was initiated 2 min after PTZ injection. Twelve mice were included in the ECG and HRV analyses (*n* = 12).

**Figure 2:**
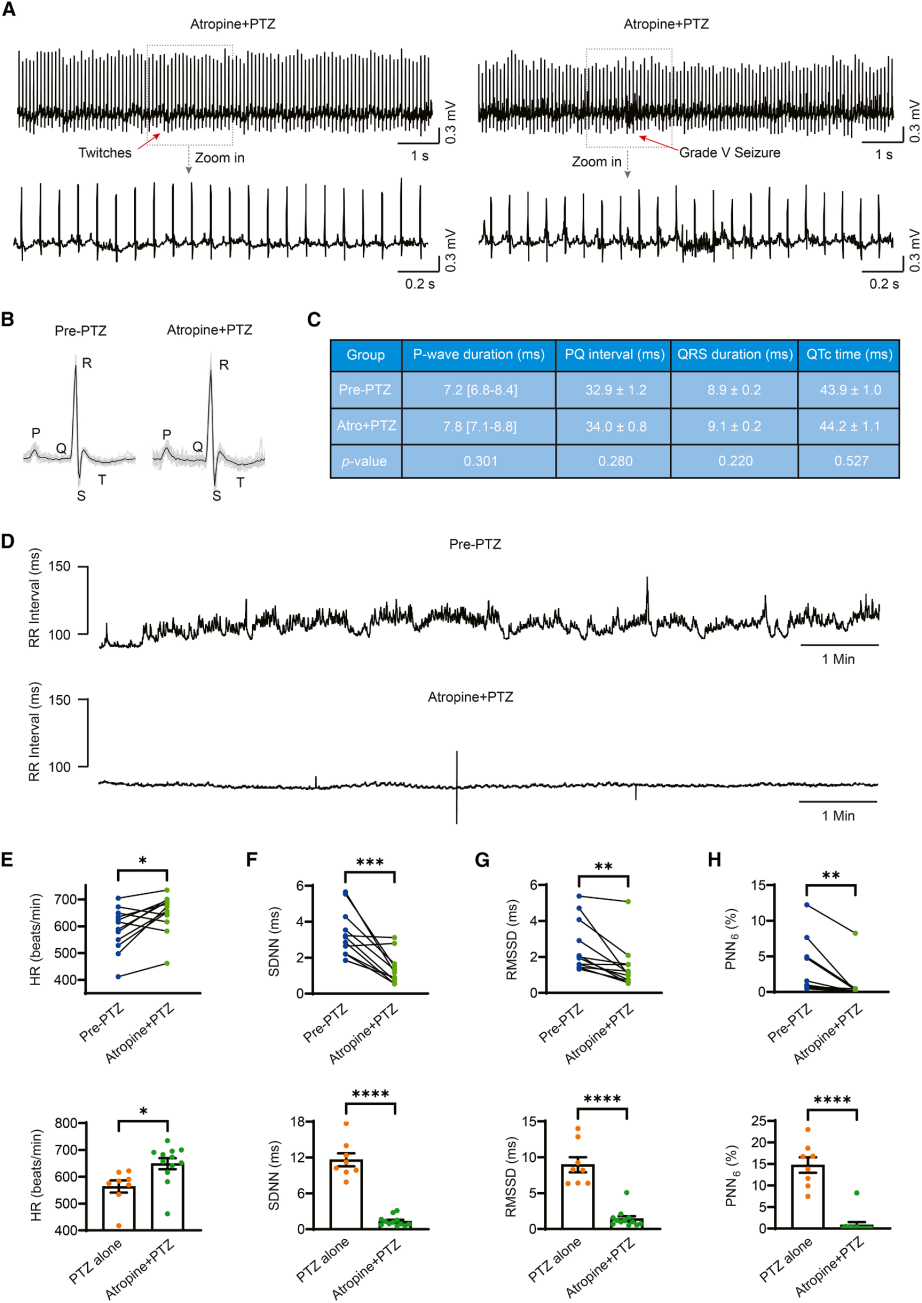
Effects of systemic muscarinic blockade on seizure-associated HRV alterations and cardiac autonomic regulation (A) Representative ECG traces from mice pretreated with atropine before PTZ administration. The left panel shows ECG activity during minor seizure-related twitches, and the right panel shows ECG during a Grade V seizure. Zoomed-in views (lower traces) highlight regular P–QRS–T wave morphology and the absence of severe arrhythmias, demonstrating that atropine pretreatment was associated with reduced seizure-associated rhythm abnormalities. (B) Representative averaged ECG waveforms from the same mouse before and after atropine + PTZ treatment, showing well-preserved P, QRS, and T wave morphology under both conditions. (C) Quantitative analysis of ECG conduction parameters. No significant differences were found in P-wave duration, PQ interval, QRS duration, or QTc time between pre-PTZ and atropine + PTZ groups (*p* > 0.05 for all; *n* = 12), indicating that atropine did not alter intrinsic myocardial conduction. P-wave duration and QRS duration were analyzed using the Wilcoxon signed-rank test, while PQ interval and QTc time were analyzed using paired t-tests. (D) R–R interval plots show that atropine pretreatment stabilized heart rate variability during seizures, suppressing irregular beat patterns observed in untreated mice. (E–H) Quantitative analysis of heart rate and time-domain HRV parameters. (E) Heart rate (HR) was significantly higher after atropine administration, consistent with the expected physiological effect of systemic muscarinic blockade. (F–H) Time-domain HRV indices were markedly reduced in atropine-treated mice: SDNN (F), RMSSD (G), and PNN_6_ (H) all decreased significantly, indicating effective attenuation of seizure-associated HRV changes. (I–L) Direct comparison between PTZ alone and atropine + PTZ groups at the post-PTZ time point. (I) HR was significantly higher in the atropine + PTZ group compared to the PTZ alone group. (J) SDNN was significantly lower in the atropine + PTZ group. (K) RMSSD and (L) PNN_6_ were both significantly reduced in the atropine + PTZ group. These results indicate that atropine pretreatment was associated with the attenuation of seizure-associated HRV alterations. Data are presented as mean ± SEM or median [IQR], depending on data distribution. Statistical significance was determined by paired t-tests or Wilcoxon signed-rank tests for within-group comparisons (C, E–H), and by Mann-Whitney U tests for between-group comparisons (I–L) because the data did not meet normality assumptions. HRV indices were evaluated with Bonferroni correction for multiple comparisons where applicable (adjusted α = 0.0167). All analyses were based on 12 mice for the atropine + PTZ group (*n* = 12) and 8 mice for the PTZ alone group (*n* = 8). ∗*p* < 0.05, ∗∗*p* < 0.01, and ∗∗∗*p* < 0.001.

As shown in Figure 2A, atropine pretreatment was associated with a marked reduction in seizure-associated rhythm abnormalities, maintaining rhythmic ECG signals throughout Grade V seizures. In contrast to untreated animals, no irregular or ectopic patterns were observed. Zoomed-in traces further confirmed rhythmic beat morphology during seizure events. Representative ECG waveforms (Figure 2B) showed no morphological differences in P, QRS, or T waves between the pre-PTZ and post-PTZ states in atropine-treated mice. Quantitative analysis (Figure 2C) revealed no significant differences in P wave duration, PQ interval, QRS duration, or QTc time between the pre- and post-treatment conditions. P wave duration was analyzed using the Wilcoxon signed-rank test, while PQ interval, QRS duration, and QTc time were analyzed using paired *t*-tests. These results indicate that no significant changes in the measured ECG conduction parameters were detected following PTZ administration in atropine-pretreated mice.

Figure 2D illustrates representative R–R interval plots before and after atropine administration. Following atropine pretreatment, R–R interval variability was markedly reduced during subsequent seizure activity. HR (Figure 2E) increased significantly after atropine administration (588 ± 23 bpm vs. 649 ± 21 bpm, *p* = 0.011, paired *t* test, power = 0.80), consistent with the expected physiological effect of systemic muscarinic blockade. Time-domain HRV analysis revealed significant reductions across all indices. SDNN decreased from 3.2 ± 0.4 ms to 1.3 ± 0.2 ms (*p* < 0.001, paired *t* test, power = 0.99; Figure 2F), while RMSSD dropped from 2.5 ± 0.4 ms to 1.4 ± 0.4 ms (*p* = 0.003, paired *t* test, power = 0.92; Figure 2G). PNN_6_ was also significantly reduced, from 0.9% [0.7%–4.8%] to 0.1% [0%–0.4%] (*p* < 0.001, Wilcoxon signed-rank test; Figure 2H). HRV indices remained statistically significant after Bonferroni correction for multiple comparisons (adjusted α = 0.0167).

To further assess the association between systemic muscarinic blockade and seizure-associated cardiac responses, we compared the post-PTZ values of HR and HRV parameters between the PTZ alone group (*n* = 8) and the atropine + PTZ group (*n* = 12). As shown in Figures 2I–2L, HR was significantly higher in the atropine + PTZ group (median 664.96 bpm [IQR 622.48–694.29]) than in the PTZ alone group (median 579.63 bpm [IQR 543.32–610.03]; Mann-Whitney U test, *p* = 0.008). In contrast, all three time-domain HRV indices were markedly lower in the atropine + PTZ group: SDNN (median 1.04 ms [IQR 0.76–1.62] vs. 10.74 ms [IQR 9.63–13.59]; Mann-Whitney U, *p* < 0.001), RMSSD (median 1.07 ms [IQR 0.70–1.58] vs. 8.24 ms [IQR 6.39–11.94]; *p* < 0.001), and PNN_6_ (median 0.078% [IQR 0.036–0.401] vs. 15.25% [IQR 10.34–18.20]; *p* < 0.001). These comparisons showed that atropine pretreatment was associated with higher HR and lower HRV indices during seizure activity.

Together, these results indicate that seizure-associated cardiac and HRV changes are sensitive to systemic muscarinic blockade. Atropine pretreatment was associated with reduced HRV changes and fewer observed rhythm abnormalities during seizures, while no significant changes in the measured ECG conduction parameters were detected.

### Cardiac autonomic regulation following repeated seizures

To evaluate whether recurrent seizures are associated with persistent alterations in cardiac autonomic regulation, 12-h daytime ECG recordings were performed after the completion of the four-week PTZ injection protocol in 11 mice that had experienced three Grade V seizures. Twelve control mice received matched saline injections across the same 4-week period and underwent ECG recordings at corresponding time points.

**Figure 3:**
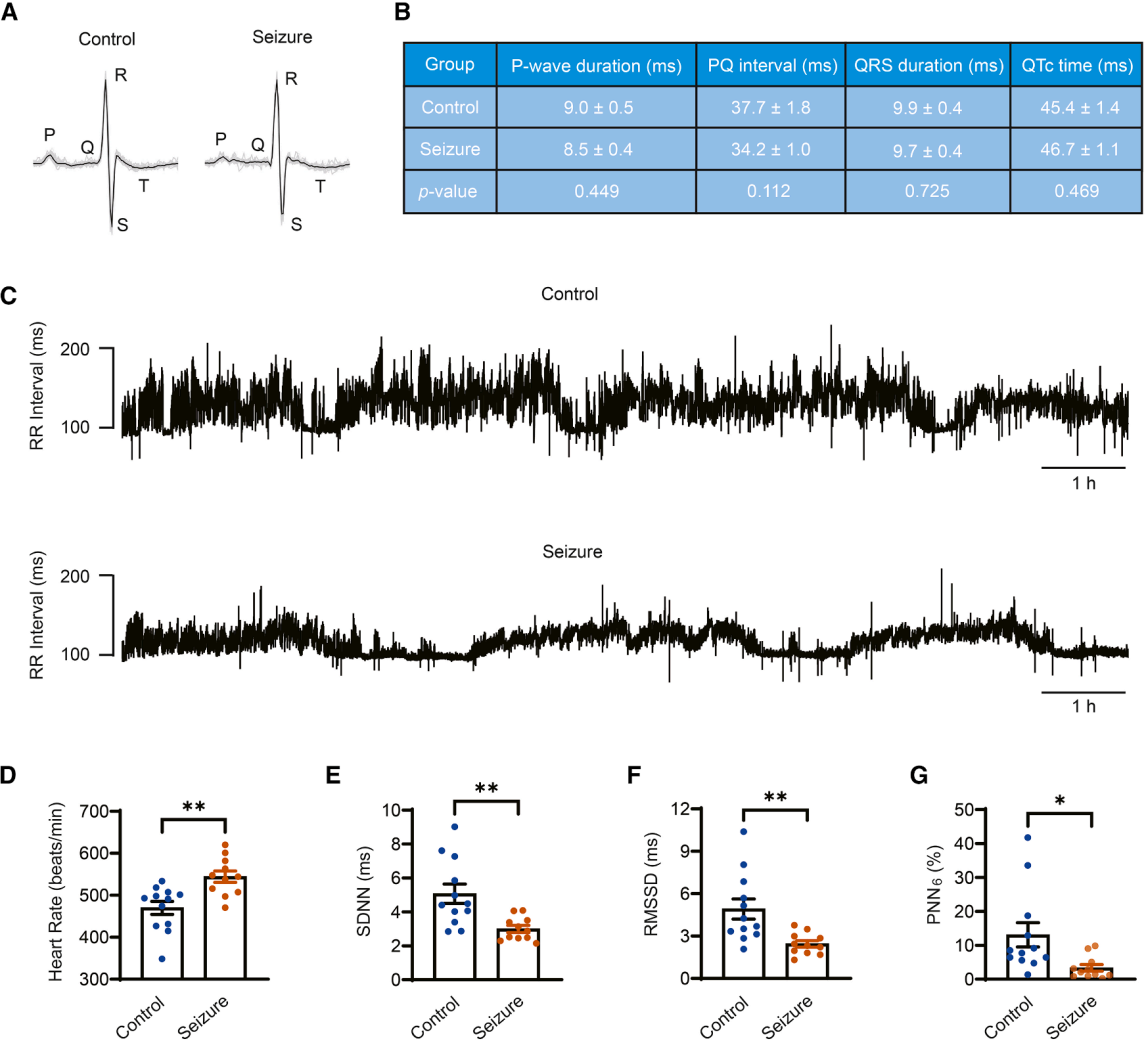
Cardiac autonomic regulation following recurrent seizures (A) Representative ECG waveforms in control (left) and seizure (right) mice show preserved P, QRS, and T wave morphology in both groups. (B) Quantitative analysis of ECG conduction parameters shows no significant differences in P-wave duration, PQ interval, QRS duration, or QTc interval between groups (*p* > 0.05), indicating that no significant differences were detected in the measured ECG conduction parameters. (C) R–R interval plots from 12-h ECG recordings reveal markedly reduced HRV in seizure mice compared to controls, indicating persistent alterations in cardiac autonomic regulation. (D–G) Quantification of heart rate and HRV parameters. (D) Heart rate was significantly elevated in seizure mice, consistent with altered autonomic regulation, possibly involving reduced parasympathetic contribution. (E–G) Time-domain HRV analysis showed significantly lower SDNN (E), RMSSD (F), and PNN_6_ (G) in seizure mice, reflecting persistent alterations in cardiac autonomic regulation. Data are presented as mean ± SEM or median [IQR] for nonparametric data. Statistical comparisons were performed using unpaired t-tests or the Mann–Whitney rank-sum test, as appropriate. HRV indices were evaluated with Bonferroni correction for multiple comparisons (adjusted α = 0.0167). Analyses were based on 12 control mice and 11 seizure mice, ∗*p* < 0.05 and ∗∗*p* < 0.01.

Representative ECG waveforms from each group are shown in Figure 3A, with clearly identifiable P, QRS, and T waves preserved in both seizure and control animals. Quantitative analysis of cardiac conduction parameters (Figure 3B) revealed no significant differences in P wave duration, PQ interval, QRS duration, or QTc interval (all *p* > 0.05). P wave duration, QRS duration, and QTc time were analyzed using unpaired *t*-tests, while PQ interval was analyzed using the Mann-Whitney rank-sum test. These findings indicate that no significant differences in the measured ECG conduction parameters were detected between seizure and control mice.

R–R interval plots from 12-h ECG recordings (Figure 3C) revealed reduced beat-to-beat variability in seizure mice, consistent with persistent alterations in cardiac autonomic regulation. Seizure mice exhibited significantly elevated HRs compared to controls (544 ± 14 bpm vs. 470 ± 15 bpm, *p* = 0.002, unpaired *t* test, power = 0.93; Figure 3D), consistent with altered autonomic regulation, possibly involving reduced parasympathetic contribution. Time-domain HRV metrics also differed significantly between groups. SDNN was significantly reduced in seizure mice (3.0 ± 0.2 ms) compared to controls (5.1 ± 0.6 ms, *p* = 0.003, unpaired *t* test, power = 0.88; Figure 3E), suggesting global autonomic dysregulation. Similarly, RMSSD, a time-domain HRV index, was markedly lower in the seizure group (2.4 ± 0.2 ms vs. 4.9 ± 0.7 ms, *p* = 0.004, unpaired *t* test, power = 0.86; Figure 3F). PNN_6_, another time-domain HRV parameter, was also significantly decreased (2.1% [0.9%–5.1%] vs. 8.1% [5.8%–17.3%], *p* = 0.006, Mann–Whitney rank-sum test; Figure 3G), consistent with persistent alterations in cardiac autonomic regulation. HRV indices remained statistically significant after Bonferroni correction for multiple comparisons (adjusted α = 0.0167).

Together, these findings indicate distinct, stage-dependent alterations in cardiac autonomic regulation across acute and repeated seizure exposure. Persistent autonomic alterations following repeated seizures may be relevant to seizure-associated cardiac complications and warrant further investigation.

### Electrophysiological properties of cardiac vagal neurons after repeated seizures

**Figure 4:**
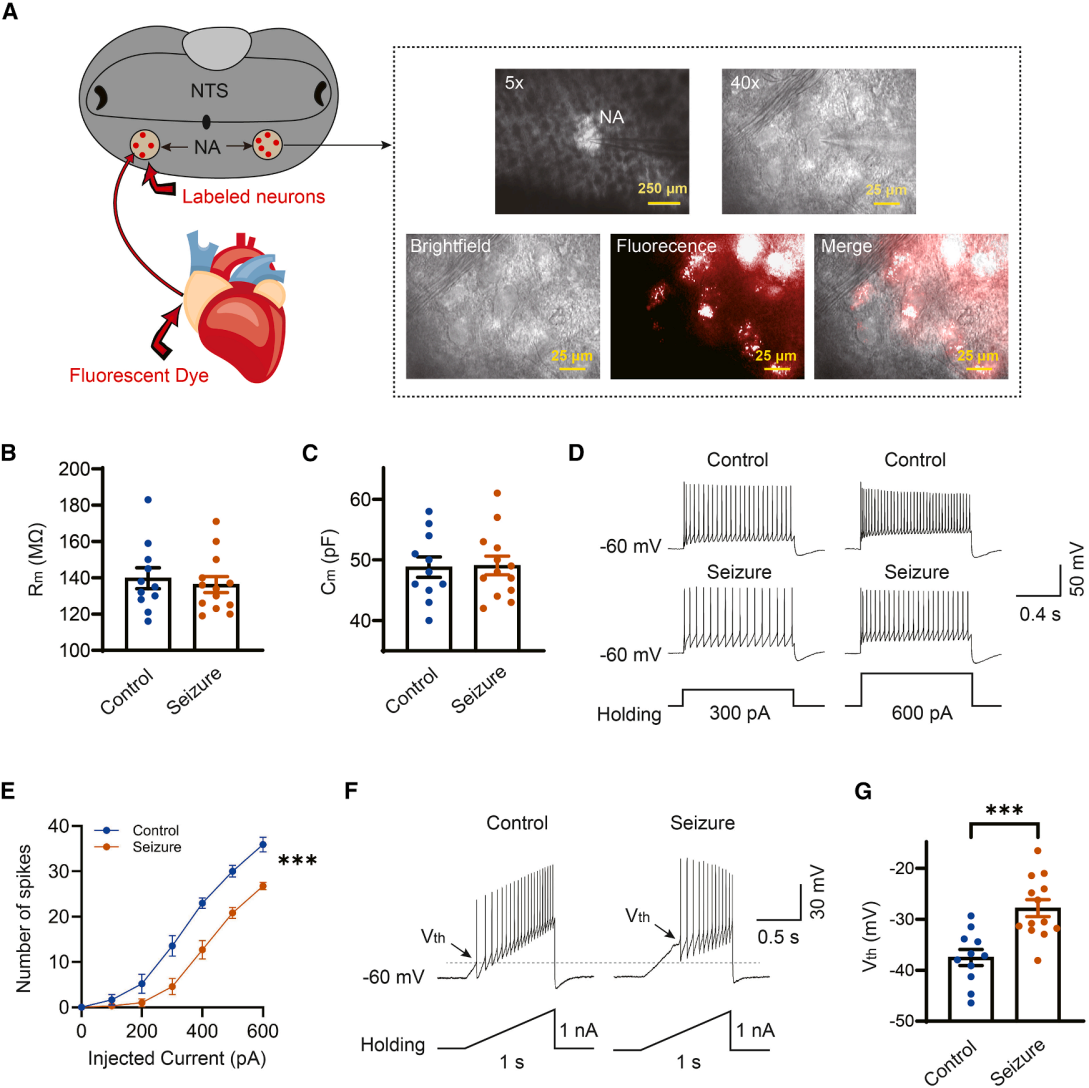
Functional changes in cardiac vagal neurons (CVNs) following repeated seizures (A) Schematic illustration of retrograde labeling of CVNs by applying DiI to the sinoatrial node. Representative low-magnification (5×) and high-magnification (40×) images of DiI-labeled neurons in the nucleus ambiguus (NA) are shown together with brightfield, fluorescence, and merged views. Scale bars, 250 μm (5× image) and 25 μm (40×, brightfield, fluorescence, and merged images). (B and C) Passive membrane properties were unchanged between groups. Input resistance (Rm, B) and membrane capacitance (Cm, C) showed no significant differences (unpaired *t* test, *p* > 0.05). (D) Representative whole-cell patch-clamp recordings of CVNs spike firing in response to square-pulse current injections (300 pA and 600 pA) in control and seizure mice. Seizure mice exhibited reduced firing frequency compared to controls. (E) Quantification of spike number as a function of injected current (100–600 pA). Seizure mice showed significantly fewer spikes across increasing current injections compared to control mice, indicating impaired excitability. ∗∗∗*p* < 0.001, two-way ANOVA with Tukey’s multiple comparisons test. (F) Representative neuronal firing responses to ramp current injections (1 nA/s) in control and seizure mice. The voltage threshold (V_th_) for action potential generation was elevated in seizure mice, suggesting reduced CVN excitability. (G) Quantification of V_th_ for action potential initiation. Seizure mice exhibited a significantly higher V_th_ compared to controls, confirming impaired neuronal excitability, ∗∗∗*p* < 0.001, unpaired *t* test. Data are presented as mean ± SEM. Analyses were based on 11 control mice and 13 seizure mice.

To investigate cellular changes potentially associated with altered cardiac autonomic regulation following repeated seizures, we examined the intrinsic excitability of CVNs in the nucleus ambiguus. Seizure model mice received intraperitoneal injections of PTZ (40 mg/kg) three times per week for four consecutive weeks, until they exhibited three Grade V seizures. Age-matched control mice were injected with an equivalent volume of saline over the same period. CVNs were retrogradely labeled by applying a fluorescent tracer to the sinoatrial node (Figure 4A). One week later, whole-cell patch-clamp recordings were performed in the nucleus ambiguus. One neuron was recorded per mouse, yielding stable recordings from 11 control mice and 13 seizure mice, which were used for all subsequent analyses.

Passive membrane properties were comparable between groups, with no significant differences in membrane resistance (Rm: 139.7 ± 5.7 MΩ in controls vs. 136.2 ± 4.4 MΩ in seizure mice, *p* = 0.633, unpaired *t* test; Figure 4B) or membrane capacitance (Cm: 48.8 ± 1.7 pF vs. 49.1 ± 1.5 pF, *p* = 0.911, unpaired *t* test; Figure 4C), indicating that baseline neuronal biophysical properties were preserved.

To evaluate intrinsic excitability, two current injection protocols were implemented. First, one-second depolarizing current steps (100–600 pA, in 100 pA increments) were applied to examine the neuronal input-output relationship. Second, a one-second ramp current injection (1 nA/s) was used to determine the voltage threshold for action potential (AP) generation. To standardize AP recordings, neurons were held at a membrane potential of −60 mV via direct current (DC) application through the recording electrode.

Representative spike firing responses to 300 pA and 600 pA square-pulse injections are presented in Figure 4D. CVNs firing increased with current amplitude in both groups (Figure 4E). However, spike output differed significantly in a current-dependent manner (two-way ANOVA, Group × Current interaction: *p* < 0.001, power = 0.961), with seizure neurons firing fewer spikes at 200–600 pA. Ramp protocols revealed that seizure neurons required greater depolarization to reach the voltage threshold for AP firing (V_th_; Figure 4F). V_th_ was significantly more depolarized in seizure mice (−27.8 ± 1.7 mV) than in controls (−37.5 ± 1.6 mV, *p* < 0.001, unpaired *t* test, power = 0.98; Figure 4G), confirming reduced excitability.

Collectively, these findings indicate that repeated seizure exposure is associated with reduced intrinsic excitability of CVNs, characterized by decreased spike output and a more depolarized AP threshold.

### Impact of recurrent seizures on myocardial electrophysiology

To assess whether recurrent seizures are associated with alterations in myocardial electrophysiological properties, optical mapping was performed to measure cardiac conduction velocity (CV) and action potential duration (APD). Optical mapping was conducted on hearts from 16 mice in total—8 seizure model mice and 8 controls—with electrical stimulation (20 pulses) applied at pacing intervals ranging from 60 to 130 ms to assess rate-dependent changes in conduction and repolarization.

**Figure 5:**
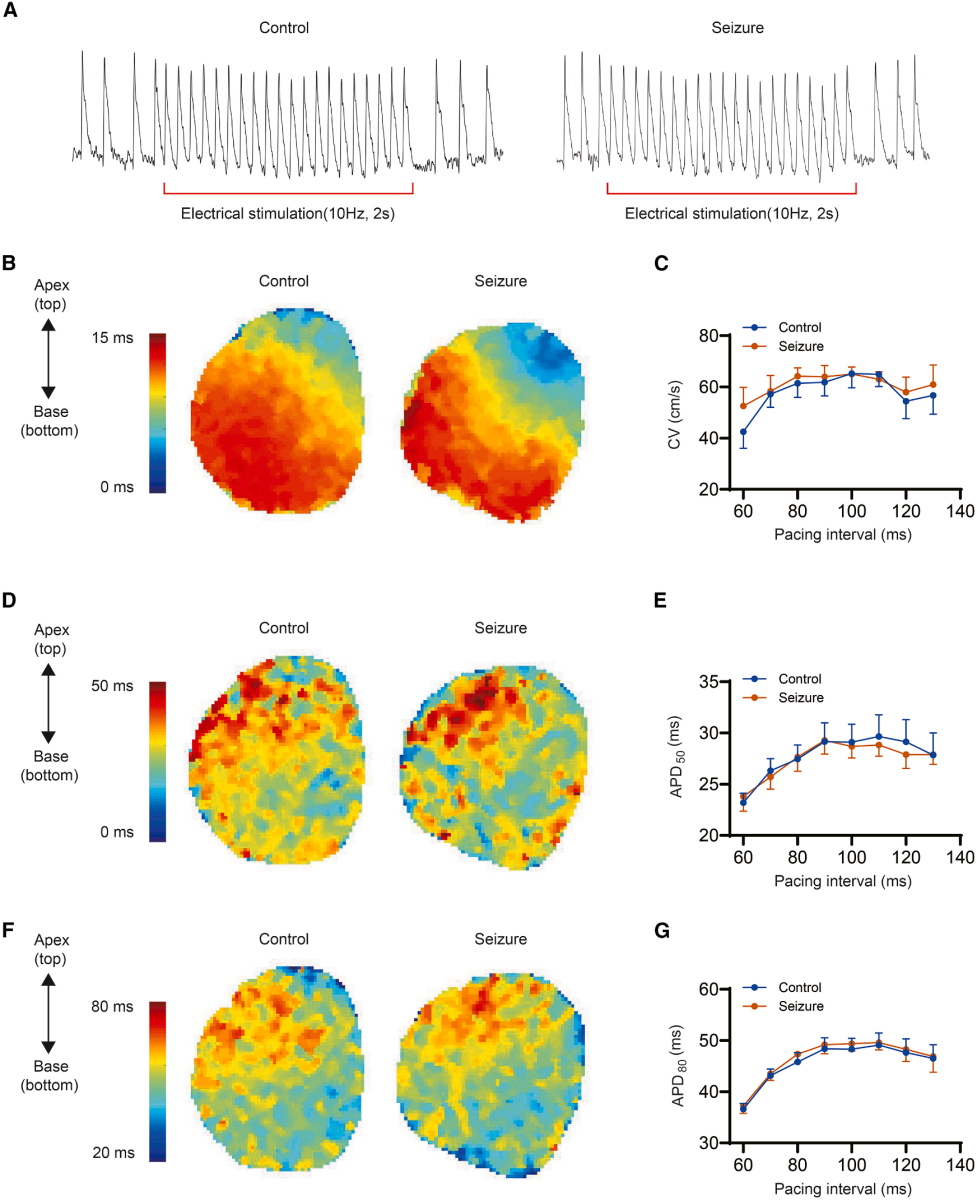
Optical mapping analysis of cardiac electrophysiology in control and seizure mice (A) Representative optical action potential traces recorded from control and seizure mice during electrical stimulation (10 Hz, 2 s). No significant differences were observed between groups. (B) Representative activation maps showing conduction velocity (CV) distribution across the heart in control and seizure mice. (C) Quantification of conduction velocity (CV) as a function of pacing interval. No significant differences were detected between control and seizure mice. (D) Representative activation maps of action potential duration at 50% repolarization (APD_50_) in control and seizure mice. (E) Quantification of APD_50_ at different pacing intervals. No significant differences were observed between control and seizure groups. (F) Representative activation maps of action potential duration at 80% repolarization (APD_80_) in control and seizure mice. (G) Quantification of APD_80_ at different pacing intervals. No significant differences were found between groups. Statistical comparisons were performed using two-way ANOVA with Tukey’s multiple comparisons test. Analyses were based on 8 control mice and 8 seizure mice.

Figure 5A displays representative optical AP traces recorded at a 100 ms pacing interval, showing comparable cardiac electrical activity between seizure and control mice. The waveform morphology remained consistent across groups, with no apparent abnormalities in response to stimulation. Figure 5B presents activation maps depicting electrical propagation across the heart. Quantitative analysis of CV (Figure 5C) showed no significant difference between seizure and control groups (*p* = 0.335, two-way ANOVA). Figures 5D and 5F illustrate the spatial distribution of APD at 50% (APD_50_) and 80% (APD_80_) repolarization, respectively. Group comparisons revealed no statistically significant differences in APD_50_ (*p* = 0.725, Figure 5E) or APD_80_ (*p* = 0.463, Figure 5G), as determined by two-way ANOVA.

Together, these findings indicate that no significant differences in CV or AP duration were detected between seizure and control hearts under the experimental conditions examined. These results provide no evidence of detectable intrinsic myocardial electrophysiological abnormalities in the measured parameters following recurrent seizures.

### Systemic muscarinic blockade and seizure-related mortality

To assess whether systemic muscarinic blockade influences seizure-related mortality, atropine was administered before PTZ-induced seizures and survival outcomes were monitored. A total of 60 mice were included in the study, comprising 41 mice receiving PTZ alone and 19 mice pre-treated with atropine (0.5 mg/kg, i.p.) 10 min before PTZ administration. Each animal was subjected to repeated PTZ injections three times per week on alternate days (i.e., every other day), with the goal of inducing three consecutive Grade V seizures. Mice were monitored throughout this period until they either completed all three seizures or succumbed during the course of the protocol. This design allowed for longitudinal assessment of seizure burden and survival across progressive convulsive episodes.

**Figure 6:**
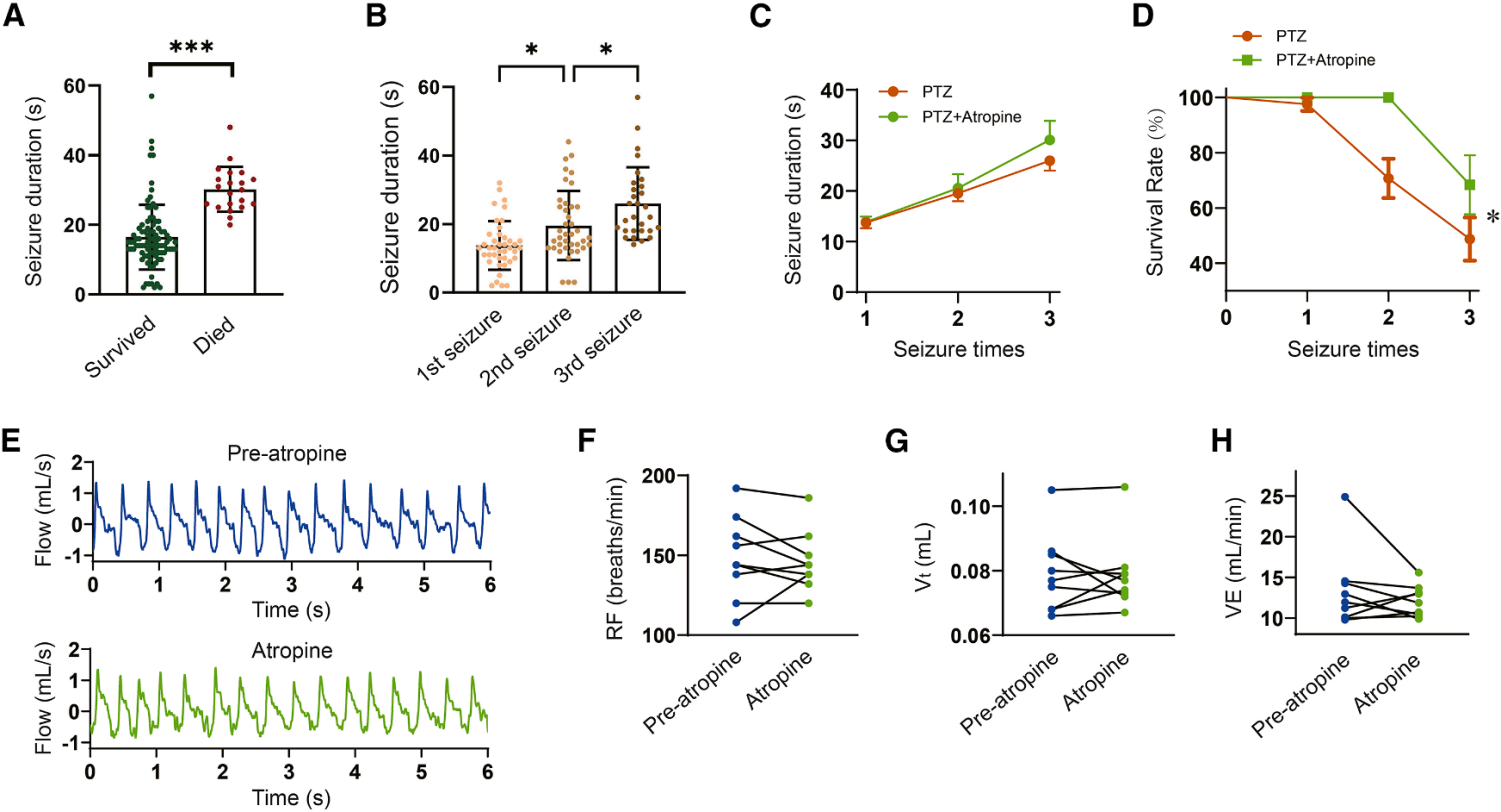
Effects of systemic muscarinic blockade on seizure duration and survival in PTZ-induced seizures (A) Comparison of seizure duration between mice that survived and those that died. Seizure duration was significantly longer in mice that did not survive. Data points represent individual seizures observed in the mice, with some mice experiencing multiple Grade V seizures. ∗∗∗*p* < 0.001, Mann-Whitney rank-sum test. (B) Seizure duration progressively increased across successive Grade V seizures. The second seizure lasted significantly longer than the first, and the third was significantly longer than the second. Each data point represents an individual Grade V seizure from a single mouse. Statistical comparisons were performed using the Kruskal–Wallis one-way ANOVA on ranks followed by Dunn’s post hoc test. ∗*p <* 0.05. (C) Atropine pretreatment did not significantly alter seizure duration across repeated Grade V seizures. Statistical comparisons were performed using two-way ANOVA with Tukey’s multiple comparisons test. Both PTZ-only and PTZ + atropine groups exhibited a similar progressive increase in seizure duration over successive episodes. (D) Survival rates in PTZ-only and PTZ + atropine groups. The bar labeled “0” represents survival prior to the first Grade V seizure. In the PTZ-only group, survival rates declined with increasing seizure episodes, whereas atropine pretreatment was associated with improved survival compared with the PTZ-only group. Survival analysis included 60 mice in total (41 PTZ-only and 19 PTZ + atropine). Seizure duration analyses in (A–C) were performed on individual Grade V seizure events, as described in the Results. (E–H) Respiratory monitoring before and after atropine administration (0.5 mg/kg, i.p.) to evaluate potential respiratory effects. (E) Representative plethysmography recordings show respiratory flow (mL/s) before (pre-atropine) and after atropine treatment. (F–H) Quantitative analysis of respiratory parameters, including respiratory frequency (RF, breaths/min), tidal volume (V_t_, mL), and minute ventilation ($\dot{V}$E, mL/min). No significant differences were observed before and after atropine administration (paired *t* test, *p* > 0.05).

All fatalities occurred during Grade V seizures and were strongly correlated with prolonged seizure duration. To evaluate this relationship, we compared the durations of individual Grade V seizures that resulted in death versus those that did not. Grade V seizures that led to death (30 [26–34] s, *n* = 21 seizures) were significantly longer than those associated with survival (15 [12–19] s, *n* = 89 seizures; *p* < 0.001, Mann-Whitney rank-sum test; Figure 6A). Here, *n* denotes the number of individual Grade V seizures, not the number of animals, as some mice experienced multiple events.

Moreover, seizure duration progressively increased across successive Grade V seizures: first (13.0 [10.0–16.5] s, *n* = 41), second (16.5 [13.0–25.0] s, *n* = 40), and third episodes (22.0 [18.0–31.5] s, *n* = 29). Statistical analysis using the Kruskal–Wallis one-way ANOVA on ranks, followed by Dunn’s post hoc test, revealed significant differences between all pairs: second vs. first (*p* = 0.010), third vs. first (*p* < 0.001), and third vs. second (*p* = 0.018; Figure 6B). These findings indicate progressively longer seizure durations across successive Grade V seizures, consistent with increasing seizure burden.

Although atropine did not significantly alter the duration of Grade V seizures (Figure 6C), it significantly improved survival rates. In the PTZ-only group (*n* = 41 mice), survival rates were 97.6% after the first seizure, 70.7% after the second, and 48.8% after the third. In contrast, atropine-treated mice (*n* = 19) exhibited 100% survival following the first and second seizures, declining to 68.4% after the third seizure (Figure 6D). Survival data were illustrated using Kaplan-Meier survival curves and evaluated by the Log rank (Mantel-Cox) test. The results showed a statistically significant improvement in survival in atropine-treated mice compared with the PTZ-only group (χ^2^ = 6.526, *p* = 0.011). These results indicate that systemic muscarinic blockade was associated with improved survival following recurrent PTZ-induced seizures. However, survival still declined with repeated seizures, underscoring the progressive vulnerability induced by cumulative seizure burden.

To confirm that the protective effect of atropine was not due to respiratory stimulation or suppression, whole-body plethysmography was performed in a separate cohort of mice (*n* = 9) before and after atropine administration (0.5 mg/kg, i.p.). Representative respiratory flow traces are shown in Figure 6E. Quantitative analysis using paired *t*-tests revealed no significant differences in respiratory frequency (RF: 148.7 ± 25.9 vs. 146.0 ± 19.0 breaths/min, *p* = 0.629), tidal volume (V_t_: 0.0789 ± 0.0122 vs. 0.0787 ± 0.0111 mL, *p* = 0.931), or minute ventilation ($\dot{V}$E: 13.30 ± 4.70 vs. 12.06 ± 1.92 mL/min, *p* = 0.332; Figures 6F–6H). These results indicate that atropine at this dose did not significantly alter baseline respiratory function under the conditions tested.

Collectively, these findings indicate that systemic muscarinic blockade was associated with improved survival following recurrent PTZ-induced seizures without detectable effects on baseline respiration. Although the relative contributions of peripheral and central muscarinic actions remain unresolved, the protective effect diminished with repeated seizures, underscoring the increasing vulnerability associated with cumulative seizure burden.

## Discussion

Epileptic seizures disrupt systemic physiological regulation, and cardiac autonomic instability is one of the most clinically relevant manifestations, contributing to morbidity and seizure-associated mortality.^5^^,^^14^^,^^15^ While peri-ictal bradyarrhythmias and HRV alterations have been documented clinically, the mechanisms underlying these changes remain unclear.^16^^,^^17^ Using a chemically induced mouse model and a multimodal approach, we show that seizure-related cardiac disturbances are associated with altered cardiac autonomic regulation in the absence of detectable intrinsic myocardial electrophysiological abnormalities. We further observed dynamic, stage-dependent alterations in cardiac autonomic regulation across acute and repeated seizures. These findings provide insight into seizure-associated cardiovascular instability and situate our observations within the broader context of autonomic dysfunction in epilepsy. Notably, severe seizure-related cardiorespiratory events, including those implicated in SUDEP, are thought to involve a terminal sequence of apnea followed by asystole.^5^^,^^9^^,^^16^

### Dynamic changes in cardiac autonomic regulation

The immediate cardiac response to a generalized convulsive seizure was characterized by bradyarrhythmias, AV block, and a marked increase in HRV indices—particularly RMSSD and PNN_6_—consistent with beat-to-beat variability during seizures. These effects were attenuated by atropine, consistent with muscarinic involvement. These acute cardiac autonomic alterations align with clinical reports of peri-ictal bradycardia and asystole^18^^,^^19^ and are consistent with previous experimental observations that PTZ-induced seizures are associated with cardiac responses sensitive to systemic muscarinic blockade. The findings suggest transient muscarinic-sensitive cardiac autonomic alterations during acute seizures.

With repeated seizures, however, we observed evidence of altered cardiac autonomic regulation, possibly involving reduced parasympathetic contribution. HRV was persistently reduced in the interictal state, and CVNs from seizure-experienced mice displayed decreased excitability and a depolarized AP threshold—consistent with altered autonomic regulation. These changes in CVN excitability may represent an adaptive response, although this interpretation remains speculative. In the context of SUDEP, such altered cardiac autonomic regulation could potentially impair the heart’s ability to respond to additional stressors (e.g., hypoxia, hypercapnia) that often accompany terminal events.^5^^,^^20^ The biphasic pattern we describe may help reconcile previously conflicting clinical reports, where some studies found reduced HRV^6^ while others suggested greater parasympathetic contribution^7^; the timing of measurement relative to seizure burden may be an important determinant.

### Cardiac autonomic regulation and myocardial electrophysiology

Several observations provide no evidence for a major intrinsic myocardial contribution to the cardiac abnormalities observed in this study. First, despite clear rhythm disturbances during seizures, ECG conduction parameters (P wave duration, PQ interval, QRS duration, QTc) remained unchanged immediately post-ictally and after four weeks of kindling. Second, optical mapping of Langendorff-perfused hearts revealed no differences in CV or AP duration between control and seizure groups, indicating preserved intrinsic myocardial electrophysiology. Third, the reduced excitability of CVNs in the nucleus ambiguus provides a cellular correlate consistent with altered cardiac autonomic regulation observed *in vivo*. Together, these findings are consistent with altered cardiac autonomic regulation in the absence of detectable intrinsic myocardial electrophysiological abnormalities under the experimental conditions examined.^9^ However, as optical mapping does not directly assess intrinsic cardiac ganglia, vagal nerve terminals, or sinoatrial node function, contributions from these peripheral structures cannot be fully excluded. This interpretation is consistent with the view that seizure-related cardiorespiratory dysfunction arises from interactions between brainstem autonomic networks and cardiac function.^5^^,^^19^

These observations are also consistent with evidence that brainstem networks coordinate both cardiac and respiratory regulation. In experimental and clinical observations, apnea often precedes cardiac dysfunction, suggesting that the disruption of brainstem respiratory centers may represent an early event, with cardiac alterations occurring secondarily.^5^^,^^9^ While our study focused on CVNs, it is plausible that neighboring respiratory neurons (e.g., in the preBötzinger complex) may undergo similar seizure-associated changes, potentially contributing to cardiorespiratory instability. Future studies that simultaneously monitor cardiac and respiratory outputs during seizures will be essential to dissect these interactions.

### Therapeutic implications

The stage-dependent alterations in cardiac autonomic regulation may have implications for therapeutic strategies. During early seizures, when muscarinic-sensitive cardiac autonomic alterations are most evident, acute pharmacological blockade with a muscarinic antagonist (e.g., atropine) was associated with reduced arrhythmia severity and improved survival (Figure 6). These findings suggest that short-acting muscarinic antagonists or closed-loop vagus nerve stimulation may warrant further investigation as potential rescue interventions for patients with frequent GTCS.

However, the protective effect of atropine appeared to diminish with repeated seizures, coinciding with the emergence of CVN hypoexcitability and prolonged seizure duration. Once vagal neurons have undergone functional changes, peripheral blockade of acetylcholine receptors may be insufficient; future interventions may need to consider mechanisms beyond peripheral muscarinic signaling. Potential strategies could include the modulation of upstream inputs to CVNs (e.g., from the nucleus tractus solitarius) or approaches aimed at restoring cardiac autonomic regulation. Moreover, given the multisystem nature of seizure-related cardiorespiratory dysfunction, combining cardiac-focused therapies with agents that enhance respiratory drive (e.g., serotonergic modulation^21^) may represent a broader approach to mitigating risk.

### Limitations of the study

Several considerations should inform the interpretation of our findings.

Model and translational considerations. The PTZ kindling model recapitulates recurrent generalized seizures—the strongest risk factor for SUDEP^18^—and enables controlled dissection of cumulative seizure burden on autonomic function.^22^ While it does not replicate all human epilepsy syndromes (e.g., focal epilepsies, Dravet syndrome),^9^^,^^12^ it reveals changes within cardiovagal circuits triggered by repeated convulsions—a core pathological element shared across many epilepsy types. In addition, only male mice were used in this study to minimize variability associated with hormonal fluctuations. Future studies including both sexes will be important to determine whether sex-specific differences influence seizure-associated autonomic regulation. Despite species differences in cardiac physiology, the core autonomic patterns we identified—dynamic stage-dependent alterations in cardiac autonomic regulation—align with clinical observations of peri-ictal bradycardia and interictal HRV reduction,^6^^,^^18^ supporting potential translational relevance. Future studies in genetic models (e.g., Scn1a^+^/^−^ mice) will determine whether similar alterations in cardiac autonomic regulation occur in syndrome-specific contexts.^9^^,^^12^

Therapeutic implications and multi-system interactions. Atropine pretreatment was associated with the attenuation of seizure-associated bradyarrhythmias and improved early survival without altering baseline respiration (Figures 6E–6H). However, its clinical utility may be limited by off-target effects, and its impact on seizure-evoked respiratory reflexes requires further evaluation. Additionally, because atropine crosses the blood–brain barrier, its protective effects may involve both peripheral cardiac and central actions; future studies using peripherally restricted muscarinic antagonists are needed to isolate cardiac-specific mechanisms. Furthermore, while our optical mapping data revealed no intrinsic myocardial abnormalities, potential contributions from intrinsic cardiac ganglia, preganglionic vagal terminals, or sinoatrial node remodeling cannot be excluded. Given that seizure-related cardiorespiratory dysfunction involves complex interactions between cardiac and respiratory control networks,^16^ future studies should explore adjunctive strategies targeting shared neuromodulatory pathways (e.g., serotonin, adenosine) for coordinated cardiorespiratory protection.^20^^,^^21^ Whether respiratory neurons (e.g., in the preBötzinger complex) undergo changes similar to those observed in CVNs remains unknown; simultaneous cardiorespiratory monitoring during seizures will be essential to dissect these interactions.^23^

Mechanistic depth and biomarkers. While our findings are consistent with an association between altered CVN function and seizure-associated cardiac abnormalities, direct manipulation of CVNs (e.g., optogenetic or chemogenetic approaches) will be required to establish causal relationships. The molecular mechanisms underlying CVN hypoexcitability—such as changes in voltage-gated ion channels, synaptic inputs, or neuroinflammation—remain to be elucidated. Transcriptomic profiling of identified CVNs may help identify potential therapeutic targets. Additionally, identifying non-invasive biomarkers of autonomic dysfunction (e.g., HRV patterns, peri-ictal respiratory parameters) may aid in risk stratification related to seizure-associated complications.^6^^,^^14^ Future studies integrating behavioral seizure severity with autonomic and electrophysiological measurements will be important to further elucidate the relationship between seizure manifestations and cardiac autonomic regulation.

## Resource availability

### Lead contact

Requests for further information and resources should be directed to and will be fulfilled by the lead contact, Junqing Sun (sunjunqing1@126.com).

### Materials availability

This study did not generate new unique reagents, experimental models, or other unique materials.

### Data and code availability

• The data reported in this paper will be shared by the lead contact upon reasonable request.
• This paper does not report original code.
• Any additional information required to reanalyze the data reported in this paper is available from the lead contact upon reasonable request.

## Acknowledgments

This work was supported by the Guangxi Science and Technology Program (grant no. GK2025GXNSFAA069809), the 10.13039/501100001809National Natural Science Foundation of China (grant nos. 32360220, 82160642), and the Sichuan Science and Technology Program (grant no. 2024JDRC0034).

## Author contributions

N.W., W.L., and J.S. conceptualized and designed the study. A.D., B.L., G.C., N.W., C.L., S.T., W.L., and J.S. were responsible for data acquisition, analysis, and interpretation. All authors contributed to drafting and/or critically revising the manuscript for important intellectual content, approved the final version for publication, and agreed to be accountable for all aspects of the work.

## Declaration of interests

The authors declare no competing interests.

## STAR★Methods

### Key resources table

**Table:** 

REAGENT or RESOURCE	SOURCE	IDENTIFIER
**Chemicals, peptides, and recombinant proteins**		

Pentylenetetrazol (PTZ)	Sigma-Aldrich	Cat# P6500
Atropine sulfate salt monohydrate	Sigma-Aldrich	Cat# A0257
1,1’-dioctadecyl 3,3,3’,3’-tetramethylindocarbocyanine perchlorate (DiI)	Sigma-Aldrich	Cat# 42364
Blebbistatin	MedChemExpress	Cat# HY-13813
RH237	AAT Bioquest	Cat# 21480
Heparin sodium	Sigma-Aldrich	Cat# H3149

**Experimental models: Organisms/strains**		

Mouse: C57BL/6J	SPF Biotechnology Co., Ltd.	RRID: IMSR_JAX:000664

**Software and algorithms**		

Kubios HRV Scientific 4.1.2	Kubios Oy	https://www.kubios.com
pCLAMP 11	Molecular Devices	https://www.moleculardevices.com
Clampfit 11.0	Molecular Devices	https://www.moleculardevices.com
ElectroMap	University of Birmingham	https://github.com/CXO531/ElectroMap
SigmaPlot 14.0	Systat Software Inc.	https://systatsoftware.com
GraphPad Prism 10.1.2	GraphPad Software	https://www.graphpad.com
Adobe Illustrator 2023	Adobe Inc.	https://www.adobe.com

**Other**		

Rechargeable telemetry device	Kardiotek Medical Biotechnology Co. Ltd.	https://www.kardiotek.cn
MultiClamp 700B Patch-Clamp Amplifier	Molecular Devices	MultiClamp 700B
Digidata 1440A Data Acquisition System	Molecular Devices	Digidata 1440A
Borosilicate Glass Capillaries	Sutter Instrument	Cat# BF150-86-10
NVSLM1 Vibrating Microtome	World Precision Instruments	Cat# NVSLM1
EMCCD Camera	Photometrics	Model: Evolve 512

### Experimental model and study participant details

#### Animals

Eight- to ten-week-old male C57BL/6J mice were obtained from SPF Biotechnology Co., Ltd. (Beijing, China). Mice were housed under specific pathogen-free (SPF) conditions in a temperature-controlled room (22 ± 2°C) with a 12-h light/12-h dark cycle and had *ad libitum* access to standard chow and water. All animal experiments were approved by the Institutional Animal Care and Use Committee of Guilin Medical University (Approval No. GLMC-IACUC-20251045) and were conducted in accordance with institutional guidelines and the National Institutes of Health Guide for the Care and Use of Laboratory Animals.

### Method details

#### Pentylenetetrazol-induced kindling epilepsy model in mice

Mice were randomly assigned to experimental and control groups. Pentylenetetrazol (PTZ) was dissolved in sterile normal saline to a final concentration of 2 mg/mL. Mice in the epilepsy model group received intraperitoneal PTZ injections (40 mg/kg) three times per week on alternate days, while control mice received an equivalent volume of saline. Seizure behavior was observed for 20 minutes after each injection and scored using Racine’s six-stage classification: Grade 0: No response; Grade I: Wet dog-like shakes, facial twitches, and chewing; Grade II: Neck muscle spasms, manifesting as nodding, and/or tail flicking; Grade III: Clonic spasms of one forelimb; Grade IV: Clonic spasms of both forelimbs accompanied by rearing; Grade V: Generalized clonic seizures with loss of balance and falling. PTZ kindling was continued until mice consistently exhibited Grade V seizures.

#### Surgical implantation of telemetry devices

All surgeries were performed under sterile conditions. Mice were anesthetized with 1.5–2% isoflurane in 100% oxygen delivered via a nose cone. The depth of anesthesia was monitored by the absence of pedal withdrawal and corneal reflexes. A rechargeable telemetry device (Kardiotek Medical Biotechnology Co., Ltd., Shanghai, China) was secured to the dorsal side of the forelimbs. The negative lead was sutured to the right upper pectoral muscle near the shoulder, while the positive lead was attached to the left xiphoid process for lead II ECG recording. Postoperative analgesia consisted of subcutaneous buprenorphine (0.05 mg/kg, twice daily) for one day. ECG recordings commenced after a 24-h postoperative recovery period, followed by time-domain HRV analysis. To ensure the stability of chronic ECG recordings, all telemetry devices were calibrated before implantation and tested for signal fidelity and battery life. ECG signals were monitored during initial recording sessions to confirm the absence of baseline drift or persistent noise. Only datasets with stable lead II signals and consistent morphology (i.e., identifiable P, QRS, and T waves) throughout the 12-hour recording were included in the final analysis. Animals with persistent signal dropout or excessive artifacts were excluded.

#### Retrograde labeling of cardiac vagal neurons with a fluorescent tracer

Retrograde labeling was performed after four weeks of epilepsy induction. Mice were anesthetized with 1.5% isoflurane, and a right thoracotomy was performed. The depth of anesthesia was monitored by the absence of pedal withdrawal and corneal reflexes. A 1 mm^2^ parafilm patch coated with the fluorescent tracer 1,1’-dioctadecyl 3,3,3’,3’-tetramethylindocarbocyanine perchlorate (DiI) was placed over the sinoatrial node, as described previously.^24^ The sinoatrial node region was identified based on established anatomical landmarks at the junction of the superior vena cava and right atrium. DiI is a lipophilic tracer that is taken up by nerve terminals at the application site and transported retrogradely to neuronal cell bodies. The patch was secured with Vetbond tissue adhesive (3M, St. Paul, MN, USA). Postoperative analgesia was administered as subcutaneous buprenorphine (0.05 mg/kg, twice daily for two days). One week was allowed for retrograde transport before further experiments.

#### Slice preparation

One week after labeling surgery, mice were anesthetized with 5% isoflurane and decapitated. The head was immediately submerged for 30 s in a slush made from high-sucrose artificial cerebrospinal fluid (aCSF) that contained (in mM) 73 NaCl, 2.5 KCl, 2 MgCl_2_, 1.25 NaH_2_PO_4_, 25 NaHCO_3_, 24 dextrose, 0.5 CaCl_2_ and 75 sucrose (300 mOSM, continuously bubbled with 95% O_2_-5% CO_2_). The brain was then exposed and submerged in ice-cold high-sucrose aCSF. Transverse brainstem slices (300 μm) were cut with a WPI NVSLM1 vibrating microtome (World Precision Instruments, Sarasota, FL). Slices were placed in a tank containing regular aCSF solution and incubated in a 35°C water bath for 30 Min, then kept at room temperature until use. Regular aCSF contained (mM) 128 NaCl, 2.5 KCl, 1 MgCl_2_, 1.25 NaH_2_PO_4_, 25 NaHCO_3_, 10 dextrose and 2 CaCl_2_ (300 mOSM, continuously bubbled with 95% O_2_-5% CO_2_).

#### Whole-cell patch clamp recording

Brainstem slices were placed in a recording chamber and continuously superfused with aCSF at room temperature. Patch electrodes (3–5 MΩ) were pulled from borosilicate glass (BF150-86-10, Sutter Instruments, USA). The pipette solution contained (in mM): 10 KCl, 128 K-gluconate, 2 MgCl_2_, 10 HEPES, 5.5 EGTA, 2 Na_2_-ATP and 0.1 CaCl_2_ (pH 7.4, adjusted with KOH).

As in our previous study,^24^ we employed a similar approach to assess neuronal excitability. To determine the intrinsic excitability of spiking responses to depolarization and action potential (AP) characteristics of cardiac vagal neurons (CVNs), two current injection protocols were performed. First, one-second depolarizing current steps (100–600 pA in 100 pA increments) were used to test general input-output relationship. Second, one-second ramp (1 nA/s) current injection was used to determine the voltage threshold for AP generation. To standardize AP recordings, neurons were depolarized to a holding potential of -60 mV by DC application through the recording electrode. Neurons were included for analysis only if they exhibited a resting membrane potential more negative than –50 mV, stable access resistance (<20 MΩ), and consistent action potential morphology without signs of depolarization block or rundown. Recordings not meeting these criteria were excluded to ensure data quality and consistency across groups. From a total of 12 and 15 attempted recordings in the control and seizure groups, respectively, 1 neuron from the control group and 2 from the seizure group were excluded due to unstable access resistance or inconsistent action potential morphology. No neurons were excluded on the basis of resting membrane potential.

Because the nucleus ambiguus (NA) is a small and localized structure, and each 300-μm brainstem slice typically contained only one intact NA region, only one cardiac vagal neuron could usually be successfully patched per mouse. Therefore, each recorded neuron represented an independent biological sample from a separate animal.

Data acquisition was controlled using the Clampex program in the pClamp11 software package (Axon Instruments, Foster City, CA). Signals were recorded using a Multi-Clamp 700B patch-clamp amplifier (Axon instruments). Responses were low-pass filtered at 2 kHz and digitized at 10 kHz with a 16-bit analog-to-digital data acquisition systems (Digidata 1440A, Axon instruments).

#### Optical mapping of membrane potential in Langendorff perfused hearts

The electrophysiological function of *ex vivo* hearts was assessed using a custom-designed optical mapping system equipped with an EMCCD camera (Evolve 512, Photometrics, Tucson, AZ, USA) as previously described.^25^ Briefly, ten minutes after an intraperitoneal injection of 100 units of heparin, the mice were anesthetized with 5% isoflurane. Following cervical dislocation, hearts were rapidly excised and Langendorff-perfused with oxygenated physiological salt solution (PSS) (containing in mM: 119 NaCl, 20 NaHCO_3_, 1.2 NaH_2_PO_4_, 4.7 KCl, 1.0 MgCl_2_, 1.3 CaCl_2_, and 10 glucose, equilibrated with 95% O2 and 5% CO2, pH 7.4) at 37°C and at a constant 2–3 ml/min flow rate for 10 min. RH237 voltage-sensitive dye (AAT Bioquest, Pleasanton, CA, USA) was loaded as a 10 μl bolus of concentration 1 mg/ml over a 10 min period. After dye loading, hearts were perfused with PSS solution containing 10 μM blebbistatin (Med Chem Express, Monmouth, Junction, NJ, USA), a myosin II inhibitor used to inhibit contractions and prevent movement artefacts during optical mapping recordings. Optical electrophysiological assessments of voltage changes used four 530 nm LEDs for RH237 excitation. Membrane potential signals were collected using a 662 nm long pass filter. The maximum camera 512 × 512 pixel^2^ area was 2-binned to optimize sampling frequency, giving a 256 × 256 pixel^2^ spatial representation of which the cardiac mapping area occupied an 83 × 83 pixel^2^ acquisition region. The work distance setting between camera and entire heart provided a distance scale of 242 × 242 μm^2^/pixel.

#### Respiratory measurement

Respiratory parameters were measured using a whole-body plethysmography (WBP) system (Shanghai Tawang Intelligent Technology Co., Ltd., China), as previously described.^26^ Each mouse was placed individually in a sealed recording chamber connected to a differential pressure transducer and data acquisition system. Baseline respiratory activity was recorded for 5 minutes under quiet resting conditions. Atropine (0.5 mg/kg, i.p.) was administered, and 10 minutes later, respiration was recorded for another 5 minutes under the same resting conditions. The system automatically converted chamber pressure fluctuations into respiratory flow signals, from which respiratory frequency (RF, breaths/min), tidal volume (Vt, mL), and minute ventilation ($\dot{V}$E, mL/min) were calculated. All analyses were performed using the manufacturer’s software, and results were expressed as means±SEM.

#### Experimental grouping and timeline

To dissect the acute and chronic effects of seizures on cardiac autonomic function while avoiding potential confounding effects of surgical intervention on central nervous system measurements, this study employed four independent cohorts of mice. Cohort 1: Acute seizure-induced ECG changes. To capture immediate cardiac responses during seizures, telemetry transmitters were implanted in mice on the day after their first Grade V seizure. Following 24 h of recovery, baseline ECG was recorded for 10 min, after which PTZ (40 mg/kg, i.p.) was administered and ECG was recorded for an additional 10 min to capture peri-ictal cardiac activity. These mice were not used in subsequent chronic experiments to avoid any influence of the implanted telemetry device or surgical recovery on central autonomic function. Cohort 2: Chronic autonomic remodeling after repeated seizures. To assess the cumulative effects of recurrent seizures, a separate group of mice received PTZ injections (40 mg/kg, i.p.) three times per week on alternate days for up to four weeks until they experienced three Grade V seizures. Telemetry devices were implanted after completion of the PTZ protocol, and 12-h daytime ECG recordings were performed 24 h after surgery. Control mice received saline injections on the same schedule and underwent identical ECG recordings. Cohort 3: *Ex vivo* cardiac electrophysiology and central vagal neuron recordings. To investigate whether repeated seizures alter intrinsic myocardial properties or central vagal excitability, independent cohorts of mice underwent the same four-week PTZ (or saline) kindling protocol as in Cohort 2, but without telemetry implantation. After completion of the protocol, mice were used for either optical mapping of Langendorff-perfused hearts or for retrograde labeling and subsequent whole-cell patch-clamp recordings of cardiac vagal neurons in the nucleus ambiguus. Cohort 4: Seizure-related mortality and pharmacological intervention. To evaluate whether systemic muscarinic blockade was associated with improved survival, a separate cohort of mice received either PTZ alone or atropine (0.5 mg/kg, i.p.) 10 min prior to each PTZ injection during the four-week kindling protocol. Survival was monitored throughout the protocol, and no ECG recordings were performed in these mice.

### Quantification and statistical analysis

ECG data were analyzed using Kubios HRV Scientific 4.1.2 (Kubios Oy, Kuopio, Finland). Prior to analysis, the data were pre-processed using a high-pass filter with a 1 Hz cutoff to remove low-frequency drift. To ensure that only sinus beats were included in the HRV analysis, non-sinus beats were excluded based on the following criteria: 1. Absence of P waves: Beats lacking identifiable P waves were excluded as non-sinus; 2. AV block: Segments showing signs of AV block, such as absent or irregular P-R intervals, were excluded. 3. Irregular R-R intervals: Beats with irregular R-R intervals, not associated with sinus rhythm, were excluded. 4. Electromyographic (EMG) interference: For prolonged Grade V seizures, we observed that EMG artifacts could result in noise that may overwhelm the ECG signal. These artifacts were excluded by visual inspection of the traces to ensure clean data for HRV analysis. For the 12-h ECG recordings obtained after completion of the repeated-seizure protocol, during which no seizures occurred, the automatic artifact correction function in Kubios HRV was sufficient to exclude non-sinus beats. Any remaining artifacts or missed beats were manually corrected by inspecting the ECG traces to ensure the data used for HRV analysis were free from interference.

To assess HRV, we calculated the following time-domain parameters: standard deviation of all normal-to-normal intervals (SDNN), it reflects the overall variability in heart rate; root mean square of successive differences between adjacent RR intervals (RMSSD), a commonly used time-domain index of HRV that is often associated with short-term autonomic modulation under physiological conditions; the percentage of successive RR intervals that differ by more than 6 ms (PNN_6_), a time-domain HRV index commonly used to assess short-term heart rate variability under sinus rhythm conditions. These parameters were used to characterize cardiac autonomic regulation and were interpreted together with pharmacological and electrophysiological findings. To minimize confounding effects of arrhythmias, HRV analysis was restricted to segments with sinus rhythm. However, given the limitations of HRV as a surrogate of autonomic activity during seizures, these measures were interpreted in conjunction with pharmacological and electrophysiological data. ECG morphological parameters, including P-wave duration, PQ interval, QRS duration, and QTc time, were determined by averaging 30 consecutive heartbeats at the animal’s average heart rate. This approach ensured consistent and reliable measurement of the ECG morphology. QTc (QT corrected) values were calculated using the Bazett formula: $QTc=\text{QT}/\sqrt{\text{RR}}$, this formula corrects the QT interval for heart rate, allowing for a standardized measure of the QT interval across varying heart rates.

The optical mapping data were processed using the ElectroMap optical mapping analysis software. This software allowed for the visualization and analysis of action potential propagation across the heart tissue. Recorded signals were spatially filtered using a 3×3 pixel Gaussian filter (σ = 1.5) to reduce noise and temporal third-order Savitzky-Golay filtering to enhance signal quality. The electrical stimulation threshold to evoke an action potential was determined initially, with 1.5 times the threshold voltage used for subsequent stimulations. Stimuli were delivered as 20 consecutive electrical stimulations with intervals ranging from 60 to 130 ms, increasing in 10 ms increments. The processed data were used to generate activation maps that display the spatiotemporal dynamics of action potentials. The results for each pixel were averaged over 20 consecutive stimuli to obtain the mean result over the entire acquisition region. Data were exported for further analysis, allowing for the extraction of relevant electrophysiological parameters such as activation time, conduction velocity, and the presence of any abnormal propagation patterns.

Patch-clamp data were processed with Clampfit 11.0 (Molecular Devices, San Jose, CA, USA). Statistical analyses were conducted using SigmaPlot 14.0 (Systat Software Inc., San Jose, CA, USA). Figures were generated in GraphPad Prism 10.1.2 (GraphPad Software, San Diego, CA, USA) and finalized using Adobe Illustrator 2023 (Adobe Systems, San Jose, CA, USA). Survival data were analyzed using Kaplan–Meier survival curves and evaluated with the Log-rank (Mantel-Cox) test to compare survival distributions between groups. Data normality was assessed using the Shapiro–Wilk test. For normally distributed data, comparisons were made using Student’s paired or unpaired t-tests, or one-way/two-way ANOVA, with appropriate post hoc analyses where applicable. For data that did not meet normality assumptions, nonparametric tests were applied, including the Mann–Whitney rank sum test for unpaired data and the Wilcoxon signed-rank test for paired data. All data are presented as mean ± SEM for parametric data or median [IQR] for nonparametric data. A *p* value < 0.05 was considered statistically significant. Where multiple comparisons were performed, *p* values were adjusted using Bonferroni correction.

Where applicable, Tukey’s multiple comparisons test was used for post hoc comparisons following two-way ANOVA, including analyses of CVN spike responses and optical mapping parameters. Dunn’s post hoc test was used following the Kruskal–Wallis test for comparisons of seizure duration across successive Grade V seizures. For the three HRV indices (SDNN, RMSSD, and PNN_6_) analyzed within the same experimental comparison in Figures 1, 2, and 3, Bonferroni correction was applied, with an adjusted significance threshold of α = 0.0167. Statistical significance in figures is indicated as follows: ∗*p* < 0.05, ∗∗*p* < 0.01, and ∗∗∗*p* < 0.001, unless otherwise specified.
